# Potential drug-drug interactions of antiretrovirals and antimicrobials detected by three databases

**DOI:** 10.1038/s41598-021-85586-8

**Published:** 2021-03-17

**Authors:** Pornpun Vivithanaporn, Teetat Kongratanapasert, Bovornpat Suriyapakorn, Pichayut Songkunlertchai, Patpicha Mongkonariyawong, Patanachai K. Limpikirati, Phisit Khemawoot

**Affiliations:** 1 Chakri Naruebodindra Medical Institute, Faculty of Medicine Ramathibodi Hospital, Mahidol University, Bang Phli, Samut Prakarn, 10540 Thailand; 2 Section for Translational Medicine, Faculty of Medicine Ramathibodi Hospital, Mahidol University, Bangkok, Thailand; 3 Department of Pharmacy Practice, Faculty of Pharmaceutical Sciences, Chulalongkorn University, Bangkok, Thailand; 4 Department of Food and Pharmaceutical Chemistry, Faculty of Pharmaceutical Sciences, Chulalongkorn University, Bangkok, Thailand; 5 Preclinical Pharmacokinetics and Interspecies Scaling for Drug Development Research Unit, Chulalongkorn University, Bangkok, Thailand

**Keywords:** Health care, Medical research

## Abstract

Standard treatment for HIV infection involves a combination of antiretrovirals. Additionally, opportunistic infections in HIV infected patients require further antimicrobial medications that might cause drug-drug interactions (DDIs). The objective of this study was to to compare the recognition of DDIs between antiretrovirals and antimicrobials by three proprietary databases and evaluate their concordance. 114 items of antiretrovirals and antimicrobials from the National List of Essential Medicines of Thailand 2018 were used in the study. However, 21 items were not recognised by Micromedex, Drugs.com, and Liverpool HIV interactions. Only 93 items were available for the detection of potential DDIs by the three databases. Potential DDIs detected from the three databases included 292 pairs. Liverpool showed the highest number of DDIs with 285 pairs compared with 259 pairs by drugs.com and 133 pairs by Micromedex. Regarding the severity classifications, Liverpool reported 10% Contraindicated; Micromedex reported 14% contraindicated and 59% major; Drugs.com reported 21% major. The Fleiss’ kappa agreements were fair to poor among the three databases, higher agreement was observed for DDIs classified as severe. This study highlights the need to harmonize the evaluation and interpretation of DDI risk in order to produce standardized information to support prescribers.

## Introduction

The human immunodeficiency virus (HIV) is a lentivirus that causes HIV infection and acquired immunodeficiency syndrome (AIDS)^1,2^. HIV infection can result in the deterioration of the immune system and lead to opportunistic infections^3,4^. Therefore, antiretroviral therapy (ART) has been introduced for HIV treatment in order to maintain the immunity of HIV infected patients^5,6^. Consequently, several drug-drug interactions (DDIs) and adverse events have been reported in the combination of ART^7,8^. These DDIs can interfere with antiretroviral efficacy, safety, and patient compliance, and thereby lead to treatment failure^9,10^. Additionally, the progression of HIV infection can lead to opportunistic infections for which further medications would be prescribed^11,12^. The addition of antimicrobials for opportunistic infections may increase the risk of DDIs^13,14^. There is a challenge in the selection of drug regimens to maximise the efficacy and minimise the toxicity of ART and antimicrobial usage in HIV patients.

Recently, electronic DDIs databases were introduced to determine potential DDIs in the prescription of HIV infected patients^15,16^. The most popular database for healthcare providers seems to be Micromedex, provided by IBM Corp., USA. This database is subject to annual subscription fees, either individually or institutionally. In contrast, Drugs.com is a free online database, favoured by patients to identify potential DDIs, as well as some pharmacists who do not subscribe to the paid database. However, several reports have mentioned that the potential DDIs detected by Micromedex and Drugs.com have low to moderate agreement^17,18^. This has resulted in differences in the identification and management of DDIs. Interestingly, there is a specialised database commonly used to determine DDIs of HIV medicines in HIV community. Liverpool HIV interaction database focused mainly on HIV drugs was selected into this study in order to determine the ability and agreement of the three electronic databases in detecting potential DDIs of antiretrovirals and antimicrobials in the national list of essential medicines of Thailand (NLEM, 2018). Furthermore, the ability and agreement of the databases was considered in order to find the most suitable information for health care providers and HIV patients to optimise drug regimens.

## Materials and methods

### Drug selection

**Figure 1:**
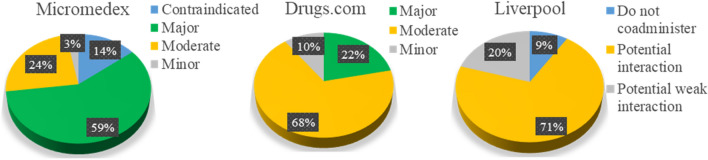
The severity of the potential DDIs characterised by Micromedex, Drugs.com, and Liverpool.

All antiretrovirals and antimicrobials were selected from the NLEM of Thailand 2018^19^. This study was conducted from 1 to 31 October 2020. Of the 645 total items, only 114 items were antiretrovirals and antimicrobials. However, 21 items were not recognised by one of the three databases, Micromedex (diethylcarbamazine, fusidic acid, protionamide, and sulbactam), Drugs.com (delamanid, diethylcarbamazine, fusidic acid, protionamide, and sulbactam), and Liverpool (artesunate, cefoperazone, cefoxitin, cefuroxime, colistimethate, dicloxacillin, fosfomycin, fusidic acid, micafungin, neomycin, netilmicin, norfloxacin, peramivir, protionamide, roxithromycin, saturated solution of potassium iodide, sulbactam, trimethoprim, and tuberculin purified protein derivative). Only 93 items were included for the determination of potential DDIs by Micromedex, Drugs.com, and Liverpool HIV interactions (Fig. 1S and Table 1S). Ethical approval and consent were not required for this study, patient assessment and confidential information were not conducted in this study.

### Databases

The Micromedex database used in this study was provided by IBM Corp., USA. This database was accessed under the copyright license of Chulalongkorn University (2020). Drugs.com is a free online database powered by four independent leading medical-information suppliers: American Society of Health-System Pharmacists, Cerner Multum, Micromedex, and Lexicomp. In total, 93 items of antiretrovirals and antimicrobials were inputted into the database on 15 October 2020, in order to determine potential DDIs among antiretrovirals and antimicrobials. Micromedex reported information on onset, severity, documentation, probable mechanism, summary, literature, clinical management, and references. Drugs.com reported potential DDIs with information on severity, description, management, and references. The Liverpool HIV interactions reported severity, quality of evidence, summary and description with references. Severity classification of potential DDIs determined by the three databases was shown in Table 1.

### Documentation of DDIs

Micromedex classifies DDIs as excellent documentation when controlled studies have established the existence of the interaction; good documentation is defined as a strong suggestion that the interaction exists, but well-controlled studies are lacking. With fair documentation, the information is poor, but pharmacologic considerations have led clinicians to suspect that the interaction exists, or the documentation is good for a pharmacologically similar drug. Drugs.com did not provide information of documentation in their database. The Liverpool documentation was reported as quality of evidence. High quality is data obtained from a randomised, controlled interaction trial with clinical or validated surrogate endpoints. Moderate is obtained from crossover or parallel, steady state pharmacokinetics (PK) study with area under curves (AUCs). Low and very low are other kinds of information, e.g., editorial comments, animal studies, case reports without AUCs.

### Data analysis

Data were entered and analysed with SPSS for windows version 22.0 (SPSS Inc., USA). Categorical variables were reported as the number and percentage. The agreement in the category of DDIs provided by the three-drug interaction databases was compared using Fleiss’ kappa. A kappa value varies between −1 and 1, with 1 indicating perfect agreement, −1 indicating perfect disagreement, and 0 indicating agreement expected by chance^20^. The interpretation of kappa values was interpreted using qualitative descriptors: intraclass correlation values > 0.80 are ‘almost perfect’; 0.61–0.80, ‘substantial’; 0.41–0.60, ‘moderate’; 0.21–0.40, ‘fair’; 0.00–0.20, ‘slight’; and < 0.00, ‘poor’^21^. The *p*-value is calculated for the kappa, with a *p*-value < 0.05 was considered statistically significant.

## Results

Among the 93 items analysed, 292 pairs of potential DDIs were detected. Micromedex reported 133 pairs, Drugs.com reported 259 pairs, and Liverpool reported 285 pairs. Of 133 pairs reported by Micromedex, 78 pairs (58.65%) were major severities. Drugs.com reported moderate severity for 177 (68.34%); meanwhile, Liverpool reported potential interactions or moderate severity for 201 (70.53%), as shown in Fig. 1. Interestingly, we found that contraindicated and major severity DDIs had a higher tendency for good and excellent documentation than moderate and minor DDIs.

**Table 1:** Severity classification of potential DDIs determined by the three databases.

Severity	Micromedex	Drugs.com	Liverpool
Contraindicated	The drugs are contraindicated for concurrent use	N/A	Do not coadminister
Major	The interaction may be life-threatening and/or require medical intervention to minimize or prevent serious adverse effects	Highly clinically significant and includes combinations that should be avoided as the risk of the interaction outweighs the benefit	N/A
Moderate	The interaction may result in exacerbation of the patient’s condition and/or require an alteration in therapy	Moderately clinically significant and includes combinations that should be avoided and used only under special circumstances	Potential interaction
Minor	The interaction would have limited clinical effects. Manifestations may include an increase in the frequency or severity of the side effects but generally would not require a major alteration in therapy	Minimally clinically significant, i.e. with minimal risk, but an alternative drug and steps taken to circumvent the interaction risk	Potential weak interaction

N/A, not applicable.

**Figure 2:**
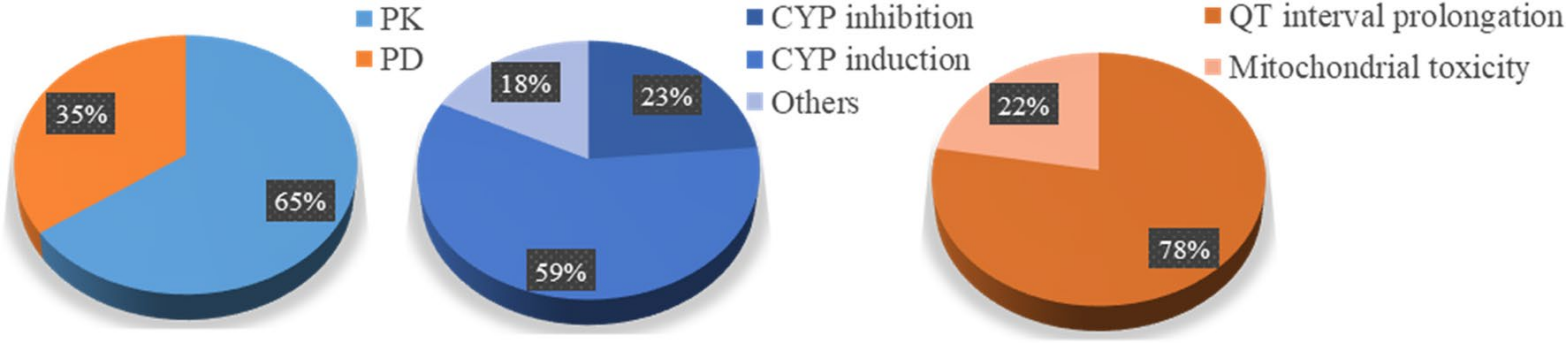
Mechanisms of serious potential DDIs determined by the three databases.

Of the 292 pairs detected by the three databases, similar severity, especially contraindicated and major, was found for 26 pairs (Table 2). For example, coadministration of atazanavir and nevirapine was characterised as contraindicated by Micromedex, major by Drugs.com, and do not coadminister by Liverpool. CYP3A4 inhibition by atazanavir and CYP3 induction by nevirapine were determined as pharmacokinetic-based mechanisms (Table 2S). Among these serious potential DDI pairs, pharmacokinetic-based DDIs had a 65% share, with CYP induction or inhibition as the main mechanisms. Meanwhile, 35% were pharmacodynamic-based DDIs, with QT-interval prolongation as the lead mechanism (Fig. 2).

**Table 2:** Drug pairs for which there is a good concordance in DDI severity grading across databases (contraindicate or major by three database).

Databases		DDIs paired lists
Micromedex—Contraindicated/major Drugs.com—Major Liverpool—Do not coadminister	1.	Atazanavir—Nevirapine
	2.	Atazanavir—Rifampicin
	3.	Darunavir—Rifampicin
	4.	Didanosine—Ribavirin
	5.	Didanosine—Stavudine
	6.	Efavirenz—Bedaquiline
	7.	Efavirenz—Erythromycin
	8.	Efavirenz—Ketoconazole
	9.	Efavirenz—Mefloquine
	10.	Efavirenz—Moxifloxacin
	11.	Efavirenz—Quinine
	12.	Efavirenz—Voriconazole
	13.	Lopinavir + Ritonavir—Bedaquiline
	14.	Lopinavir + Ritonavir—Chloroquine
	15.	Lopinavir + Ritonavir—Efavirenz
	16.	Lopinavir + Ritonavir—Moxifloxacin
	17.	Lopinavir + Ritonavir—Rifampicin
	18.	Nevirapine—Rifampicin
	19.	Rilpivirine—Efavirenz
	20.	Rilpivirine—Moxifloxacin
	21.	Rilpivirine—Rifampicin
	22.	Ritonavir—Ketoconazole
	23.	Ritonavir—Quinine
	24.	Ritonavir—Rifampicin
	25.	Ritonavir—Voriconazole
	26.	Tenofovir disoproxil fumarate—Didanosine

Surprisingly, there are a large number of DDIs with poor concordance in severity grading across the three databases, e.g., minor or none by Micromedex and Drugs.com versus do not administer or potential interactions by Liverpool database (76 DDI pairs). For example, coadministration of Atazanavir and azithromycin was reported as minor or none by Micromedex and Drugs.com, meanwhile Liverpool database determined as potential interaction. This discordance might be due to the quality of evidence of this potential DDI was very low, and coadministration has never been studied. Both compounds could develop QT prolongation and atazanavir shows inhibitory effect on efflux transporters that responsible for azithromycin elimination. Therefore, atazanavir could potentially increase azithromycin exposure, leading to the increased risk for QT prolongation. This information might lead to the discrepancy in potential DDI classification among databases (Table 3).

**Table 3:** Drugs pairs with poor concordance in DDI severity grading across databases.

Databases		DDIs paired lists
MicromedexMinor or None Drugs.com—Minor or None Liverpool—Do not coadminister or Potential interaction	1.	Abacavir—Peginterferon alfa-2a
	2.	Abacavir—Peginterferon alfa-2b
	3.	Atazanavir—Azithromycin
	4.	Atazanavir—Ciprofloxacin
	5.	Atazanavir—Mebendazole
	6.	Atazanavir—Ofloxacin
	7.	Atazanavir—Pentamidine
	8.	Atazanavir—Praziquantel
	9.	Atazanavir—Primaquine
	10.	Atazanavir—Ribavirin
	11.	Darunavir—Clindamycin
	12.	Darunavir—Mebendazole
	13.	Darunavir—Metronidazole
	14.	Darunavir—Moxifloxacin
	15.	Darunavir—Voriconazole
	16.	Didanosine—Amikacin
	17.	Didanosine—Amphotericin B
	18.	Didanosine—Capreomycin
	19.	Didanosine—Cidofovir
	20.	Didanosine—Doxycycline
	21.	Didanosine—Flucytosine
	22.	Didanosine—Lamivudine
	23.	Didanosine—Para-aminosalicylic acid
	24.	Didanosine—Pyrazinamide
	25.	Didanosine—Pyrimethamine
	26.	Didanosine—Tetracycline
	27.	Efavirenz—Clindamycin
	28.	Efavirenz—Doxycycline
	29.	Efavirenz—Primaquine
	30.	Efavirenz—Sofosbuvir + Ledipasvir
	31.	Emtricitabine—Flucytosine
	32.	Emtricitabine—Peginterferon alfa-2a
	33.	Emtricitabine—Peginterferon alfa-2b
	34.	Emtricitabine—Sulfadiazine
	35.	Lamivudine—Flucytosine
	36.	Lamivudine—Peginterferon alfa-2a
	37.	Lamivudine—Peginterferon alfa-2b
	38.	Lamivudine—Sulfadiazine
	39.	Lopinavir + Ritonavir—Praziquantel
	40.	Lopinavir + Ritonavir—Sofosbuvir + Ledipasvir
	41.	Nevirapine—Clindamycin
	42.	Nevirapine—Doxycycline
	43.	Nevirapine—Griseofulvin
	44.	Nevirapine—Primaquine
	45.	Rilpivirine—Didanosine
	46.	Rilpivirine—Griseofulvin
	47.	Ritonavir—Darunavir
	48.	Ritonavir—Mefloquine
	49.	Ritonavir—Praziquantel
	50.	Ritonavir—Sofosbuvir + Ledipasvir
	51.	Stavudine—Amikacin
	52.	Stavudine—Amphotericin B
	53.	Stavudine—Ampicillin
	54.	Stavudine—Capreomycin
	55.	Stavudine—Cefotaxime
	56.	Stavudine—Cephalexin
	57.	Stavudine—Cidofovir
	58.	Stavudine—Flucytosine
	59.	Stavudine—Para-aminosalicylic acid
	60.	Stavudine—Pentamidine
	61.	Stavudine—Sulfadiazine
	62.	Stavudine—Sulfamethoxazole + Trimethoprim
	63.	Tenofovir—Piperacillin + Tazobactam
	64.	Tenofovir disoproxil fumarate—Clarithromycin
	65.	Tenofovir disoproxil fumarate—Itraconazole
	66.	Tenofovir disoproxil fumarate—Ketoconazole
	67.	Tenofovir disoproxil fumarate—Peginterferon alfa-2a
	68.	Tenofovir disoproxil fumarate—Peginterferon alfa-2b
	69.	Tenofovir disoproxil fumarate—Sulfadiazine
	70.	Zidovudine—Albendazole
	71.	Zidovudine—Mebendazole
	72.	Zidovudine—Nitrofurantoin
	73.	Zidovudine—Primaquine
	74.	Zidovudine—Sulfadiazine
	75.	Zidovudine—Sulfamethoxazole + Trimethoprim
	76.	Zidovudine—Vancomycin

The agreement among the severity reports of the three databases, as determined by Fleiss’ kappa value, was 0.129 (0.127 to 0.132, *p* < 0.001), which was considered to be a slight agreement among the three databases, as detailed in Table 4. While the agreement between two databases, Micromedex and Drugs.com, for the determination of all potential DDIs was 0.160 (0.156 to 0.165, *p* < 0.001), indicated slight agreement. Higher severity DDIs showed tendency for better agreement among databases.

**Table 4:** Agreement among three drug-interaction databases.

Category	Kappa	95% CI	*p*-value	Strength of agreement
Minor	− 0.010	− 0.013 to − 0.007	0.84	Poor
Moderate	0.243	0.240 to 0.246	< 0.001	Fair
Major	− 0.090	− 0.093 to − 0.087	0.072	Poor
Contraindicated	0.349	0.346 to 0.352	< 0.001	Fair

## Discussion

Antiretroviral combinations are a standard regimen for the treatment of HIV infected patients. Combination of antiretroviral drugs increase antiretroviral activity and reduces the risk of acquiring resistance^22,23^. Antiretroviral drugs have a high potential for drug-drug interactions which can occur with comedications used to treat for instance opportunistic infections^24,25^. In recent years, several online databases have been developed to determine potential DDIs. The present study selected three common databases, including Micromedex, Drugs.com, and Liverpool database, to determine the potential DDIs of antiretrovirals and antimicrobials available in the NLEM of Thailand 2018. Among 93 medicines included in this study, there were approximately 8,000 pairs of medicines. However, only 3–4% (292/8,000 pairs) could develop potential DDIs as determined by the three databases, compared with 18–20% (1,285/7,000 pairs) of potential DDIs in metabolic syndrome medications described in our previous report^18^. Even though the percentage of potential DDIs among antiretrovirals and antimicrobials was lower than that found for metabolic syndrome medications, the degree of severity seemed to be higher. Approximately 70% (97/133 pairs) of potential DDIs among antiretrovirals and antimicrobials were classified as contraindicated or major by Micromedex. Meanwhile, only 20% (155/724 pairs) of potential DDIs in metabolic syndrome medications were determined as contraindicated or major by Micromedex. Therefore, potential DDIs of antiretrovirals and antimicrobials should be taken into consideration regarding the clinical impact related to both treatment failure and serious adverse events.

Among 292 pairs of potential DDIs, there were 26 pairs of major concerns. The three databases reported that these 26 pairs are contraindicated or major DDIs. Most of these serious DDIs have mechanisms related to CYP induction or inhibition and QT interval prolongation. Among the antiretroviral combinations in ART, protease inhibitors seemed to be the most troublesome class of medicines with moderate to strong CYP inhibition, and a less degree of CYP induction. For example, ritonavir is an enzyme inducer of CYP1A2 and CYP2C; meanwhile, it is an enzyme inhibitor of CYP3A4 and CYP2D6^26^. Induction and inhibition of CYPs could interfere with the metabolism of commonly available medicines because CYPs are the major drug-metabolising enzymes; approximately 50% of the top 200 prescribed drugs were biotransformed by CYPs^27^. Besides, non-nucleotide reverse transcriptase inhibitors such as Efavirenz is a moderate inducer of CYP3A4 whereas nevirapine is a moderate-weak inducer, which might reduce exposure of concomitant drugs if they are a substrate of CYP3A4. Avoidance of CYP overlapping antiretroviral combinations or therapeutic drug monitoring would be useful to prevent further complications with antiretroviral usage in HIV patients.

In the case of bacterial infections, the addition of antibacterials to ART seems to raise precaution for two groups of medicines, i.e. macrolides and quinolones. Erythromycin and clarithromycin have moderate to strong CYP inhibitory properties, and a class effect with QT-interval prolongation, which might lead to cardiac arrhythmia. Quinolones also showed similar properties to macrolides, especially CYP inhibition and QT-interval prolongation^28^. Therefore, coadministration of these antibacterials should be exempted from concomitant use with protease inhibitors and non-nucleotide reverse transcriptase inhibitors. Beta-lactam antibacterials seemed to be the better choice for coadministration with protease inhibitors or non-nucleotide reverse transcriptase inhibitors. Because most beta-lactams have good water solubility and minimal CYP biotransformation, they are mainly excreted via the kidney. Interestingly, the standard treatment of mycobacterial infections, e.g., tuberculosis, needs a combination of three to five antimycobacterials to eradicate the pathogen and prevent long-term resistance. Rifampicin is a potent CYP inducer; meanwhile, ethambutol is a CYP inhibitor; these two drugs are the backbone of the standard treatment for tuberculosis in Thailand^29^. It is based on the fact that enzyme inhibition could occur immediately after administration, whereas enzyme induction need more time for protein expression. Therefore, rifampicin is likely to have delay and stronger induction effect compared with rapidly inhibitory effect of ethambutol. In this case, therapeutic drug monitoring would be useful if adverse events or treatment failure were observed from any suspected drugs.

Fungal infections in HIV patients can range from mild to severe. Azole derivatives seemed to be the most popular antifungals due to good safety profiles and convenient dosage forms, i.e. intravenous, oral, and topical preparations. These azole antifungals have excellent inhibitory properties to fungal CYP450s and to a lesser extent to human CYP450s. Itraconazole and posaconazole are more potent inhibitors of human CYP3A4 compared with fluconazole and voriconazole. Also, fluconazole and voriconazole are strong inhibitors of CYP2C9 and CYP2C19. Therefore, coadministration of azole antifungals and some protease inhibitors or non-nucleotide reverse transcriptase inhibitors would be careful with potential DDIs. Alternative antifungals against specific pathogens or therapeutic drug monitoring would be possible choices to manage the potential DDIs between antifungals and antiretrovirals.

Optimistically, the majority of antivirals against opportunistic infections are nucleoside analogues e.g., ganciclovir, acyclovir. These compounds are endowed with fairly high water solubility owing to the heterocyclic base and sugar in their structures. There is thus no need for CYP biotransformation and these drugs are predominantly eliminated through renal excretion. Therefore, potential DDIs, especially pharmacokinetic-based interactions, are not likely to arise via CYP inhibition or induction. In addition, anti-hepatitis B and C drugs also show similar results compared with those from the aforementioned antivirals. Tenofovir and sofosbuvir are nucleotide analogues that contain base, sugar and phosphate moieties in their structures. Actually, high water solubility can be expected in tandem with minimal biotransformation via hepatic CYP enzymes, but these antiviral agents might be substrate of some transporters during excretion. Organic anion transporters are important for renal tubular secretion of guanine containing antivirals, e.g., ganciclovir, acyclovir. In addition, sofosbuvir and other NS5A inhibitors were reported as substrates of some efflux transporters, e.g., p-glycoprotein. These drugs can be victims of DDIs via inhibition or induction of drug transporters. Further information in antiviral DDIs via transmembrane transports are urgently required for database development.

Management of potential DDIs among antiretrovirals and antimicrobials is a crucial step in HIV treatment. The three databases were able to detect serious DDIs with contraindicated and major severity. Alternative drugs with different pharmacokinetic or pharmacodynamic pathways seem to be appropriate options^30^. Other possible management involves close monitoring and dose adjustment to prevent adverse events^31^. Therefore, patient consultation and physician communication should be stressed to improve awareness of significant potential DDIs. These actions would be useful to prevent treatment failure and improve patient compliance with HIV treatment. One limitation of this study is the inclusion of antiretrovirals and antimicrobials, only drugs on the NLEM of Thailand 2018 were included, which did not cover all available drugs in each class. Additionally, the three databases might update information frequently, so during different search periods might provide different results. The difference between the drug interaction databases might be caused by the use of different references, unique rating priorities, and display formats^32^. Difference sources of references could be resulting in different severity classification and inconsistency among databases has been reported^33^. Not finding a comedication does not mean that there is no risk of DDIs. Combination of antibacterials or antivirals with similar metabolic pathways and toxicity meachnisms could lead to serious potential DDIs. Careful determination of potential DDIs by multiple databases and consideration for their agreement is necessary. Currently, there are no standard criteria for defining potential DDIs, resulting in complex interpretation of the results^34,35^. Therefore, a method to standardise DDIs information is required and the use of more than one database may be necessary.

## Conclusion

A large number of potential DDIs were detected among antiretrovirals and antimicrobials used for opportunistic infection in the National List of Essential Medicines of Thailand 2018. Liverpool database showed the highest number of potential DDIs. DDIs with high severity as classified by contraindicated or major had better agreement value among the three databases. CYP induction or inhibition and QT prolongation were leading causes of serious potential DDIs. The observed poor agreement among databases highlights the need to harmonize the evaluation and interpretation of DDI risk in order to produce standardized information to support prescribers.

## Supplementary Information

Supplementary Information

## Abbreviations

AIDS: Acquired immunodeficiency syndrome
ART: Antiretroviral therapy
AUCs: Area under curves
CYPs: Cytochrome P450s
DDIs: Drug-drug interactions
HIV: Human immunodeficiency virus
NLEM: National list of essential medicine
PD: Pharmacodynamics
PK: Pharmacokinetics
UGTs: Uridine glucuronosyl transferases
