# Supplementary material for: Potential drug-drug interactions of antiretrovirals and antimicrobials detected by three databases
Source: Sci Rep. 2021 Mar 17;11:6089. doi: 10.1038/s41598-021-85586-8 (PMC7971054; doi:10.1038/s41598-021-85586-8)
Supplement: Supplementary file 1 — Supplementary Information [file 41598_2021_85586_MOESM1_ESM.docx]

**Potential drug-drug interactions of antiretrovirals and antimicrobials detected by three databases**

Pornpun Vivithanaporn^1^, Teetat Kongratanapasert^2^, Bovornpat Suriyapakorn^3^, Pichayut Songkunlertchai^3^, Patpicha Mongkonariyawong^3^, Patanachai K. Limpikirati^4^, Phisit Khemawoot^1*,5^

**Affiliations**

1 Chakri Naruebodindra Medical Institute, Faculty of Medicine Ramathibodi Hospital, Mahidol University, Samutprakarn, Thailand

2 Section for Translational Medicine, Faculty of Medicine Ramathibodi Hospital, Mahidol University, Bangkok, Thailand

3 Department of Pharmacy Practice, Faculty of Pharmaceutical Sciences, Chulalongkorn University, Bangkok, Thailand

4 Department of Food and Pharmaceutical Chemistry, Faculty of Pharmaceutical Sciences, Chulalongkorn University, Bangkok, Thailand

5 Preclinical Pharmacokinetics and Interspecies Scaling for Drug Development Research Unit, Chulalongkorn University, Bangkok, Thailand

***Corresponding author**

Phisit Khemawoot, Ph.D.

Chakri Naruebodindra Medical Institute, Faculty of Medicine Ramathibodi Hospital, Mahidol University, Bang Phli, Samut Prakarn 10540, Thailand

Tel.: +66 28395161; Fax: +66 28395161 E-mail address: phisit.khe@mahidol.ac.th

Antiretrovirals and antimicrobials,

114 items

Available drugs for the detection of potential DDIs,

93 items

Unavailable drugs in Micromedex, Drugs.com, or Liverpool database,

21 items

Potential DDIs detected from Micromedex, Drugs.com, or Liverpool database,

292 pairs

Total drug lists from NLEM 2018,

645 items

Exclusion

**Figure 1S. Drug selection and potential DDIs determined by Micromedex, Drugs.com, and Liverpool database.**

**Table 1S. Antiretroviral and antimicrobial lists for the determination of potential DDIs.**

| Drug Class | Drug Lists |
| --- | --- |
| Penicillins and beta-lactamase inhibitors | 1. Amoxicillin |
|  | 2. Ampicillin |
|  | 3. Cloxacillin |
|  | 4. Penicillin V potassium |
|  | 5. Penicillin G sodium |
|  | 6. Penicillin G benzathine |
|  | 7. Penicillin G procaine |
|  | 8. Amoxicillin + Clavulanate |
|  | 9. Piperacillin + Tazobactam |
| Cephalosporins, cephamycins and other beta-lactams | 10. Cephalexin |
|  | 11. Cefazolin |
|  | 12. Cefotaxime |
|  | 13. Ceftriaxone |
|  | 14. Ceftazidime |
|  | 15. Cefixime |
|  | 16. Ertapenem |
|  | 17. Imipenem + Cilastatin |
|  | 18. Meropenem |
| Tetracyclines | 19. Doxycycline |
|  | 20. Tetracycline |
| Aminoglycosides | 21. Gentamicin |
|  | 22. Amikacin |
| Macrolides | 23. Erythromycin |
|  | 24. Azithromycin |
|  | 25. Clarithromycin |
| Quinolones | 26. Ofloxacin |
|  | 27. Ciprofloxacin |
|  | 28. Levofloxacin |
| Other antibacterials | 29. Chloramphenicol |
|  | 30. Metronidazole |
|  | 31. Clindamycin |
|  | 32. Nitrofurantoin |
|  | 33. Vancomycin |
|  | 34. Linezolid |
| Sulfonamides and trimethoprim | 35. Sulfamethoxazole + Trimethoprim |
| Antituberculous drugs | 36. Ethambutol |
|  | 37. Isoniazid |
|  | 38. Pyrazinamide |
|  | 39. Rifampicin |
|  | 40. Streptomycin |
|  | 41. Cycloserine |
|  | 42. Ethionamide  43. Kanamycin |
|  | 44. Para-aminosalicylic acid |
|  | 45. Bedaquiline |
|  | 46. Capreomycin |
|  | 47. Moxifloxacin |
| Antileprotic drugs | 48. Dapsone |
| Antimalarials | 49. Chloroquine |
|  | 50. Primaquine |
|  | 51. Quinine |
|  | 52. Mefloquine |
| Other antiprotozoal drugs | 53. Pyrimethamine |
|  | 54. Sulfadiazine |
|  | 55. Pentamidine |
| Antifungal drugs | 56. Fluconazole |
|  | 57. Griseofulvin |
|  | 58. Ketoconazole |
|  | 59. Nystatin |
|  | 60. Itraconazole |
|  | 61. Amphotericin B |
|  | 62. Flucytosine |
|  | 63. Voriconazole |
| Antiviral drugs | 64. Acyclovir |
|  | 65. Cidofovir |
|  | 66. Ganciclovir |
|  | 67. Oseltamivir |
| Anti-hepatitis drugs | 68. Entecavir |
|  | 69. Peginterferon alfa-2a |
|  | 70. Peginterferon alfa-2b |
|  | 71. Ribavirin |
|  | 72. Sofosbuvir |
|  | 73. Sofosbuvir + Ledipasvir |
| Antihelmintics | 74. Albendazole |
|  | 75. Mebendazole  76. Niclosamide |
|  | 77. Praziquantel |
|  | 78. Ivermectin |
| Integrase inhibitors | 79. Raltegravir |
| Non-nucleoside reverse transcriptase inhibitors | 80. Efavirenz |
|  | 81. Nevirapine |
|  | 82. Rilpivirine |
| Nucleoside/nucleotide reverse transcriptase inhibitors | 83. Abacavir |
|  | 84. Didanosine |
|  | 85. Emtricitabine |
|  | 86. Lamivudine |
|  | 87. Stavudine |
|  | 88. Tenofovir disoproxil fumarate |
|  | 89. Zidovudine |
| Protease inhibitors | 90. Ritonavir |
|  | 91. Atazanavir |
|  | 92. Darunavir |
|  | 93. Lopinavir + Ritonavir |

**Table 2S. Classification of major DDI mechanisms among antiretrovirals and antimicrobials.**

|  | DDIs paired lists | Mechanism |
| --- | --- | --- |
|  | Atazanavir - Nevirapine | PK - CYP3A4 inhibition by Atazanavir, CYP3A4 induction by Nevirapine |
|  | Atazanavir - Rifampicin | PK - CYP3A induction by Rifampicin |
|  | Darunavir - Rifampicin | PK - CYP3A induction by Rifampicin |
|  | Didanosine - Ribavirin | PD - Increase risk of mitochondrial toxicities |
|  | Didanosine - Stavudine | PD - Increase risk of certain toxicities related to mitochondrial damage |
|  | Efavirenz - Bedaquiline | PK - CYP3A4 induction by Efavirenz |
|  | Efavirenz - Erythromycin | PD - Addition of QT-interval prolongation |
|  | Efavirenz - Ketoconazole | PK - CYP3A4 induction by Efavirenz |
|  | Efavirenz - Mefloquine | PD - Addition of QT-interval prolongation |
|  | Efavirenz - Moxifloxacin | PD - Addition of QT-interval prolongation |
|  | Efavirenz - Quinine | PD - Addition of QT-interval prolongation |
|  | Efavirenz - Voriconazole | PK - CYP3A induction by Efavirenz, CYP3A inhibition by Voriconazole |
|  | Lopinavir + Ritonavir - Bedaquiline | PK - CYP3A4 inhibition |
|  | Lopinavir + Ritonavir - Chloroquine | PD - Addition of QT-interval prolongation |
|  | Lopinavir + Ritonavir - Efavirenz | PK - CYP3A induction by Efavirenz |
|  | Lopinavir + Ritonavir - Moxifloxacin | PD - Addition of QT-interval prolongation |
|  | Lopinavir + Ritonavir - Rifampicin | PK - CYP3A4 induction by Rifampicin |
|  | Nevirapine - Rifampicin | PK - CYP3A4 induction by Rifampicin |
|  | Rilpivirine - Efavirenz | PK- Decrease Rilpivirine serum concentration |
|  | Rilpivirine - Moxifloxacin | PD - Addition of QT-interval prolongation |
|  | Rilpivirine - Rifampicin | PK - CYP3A induction by Rifampicin |
|  | Ritonavir - Ketoconazole | PK - CYP3A4 inhibition |
|  | Ritonavir - Quinine | PK - CYP3A4 inhibition by Ritonavir |
|  | Ritonavir - Rifampicin | PK - CYP induction by Rifampicin |
|  | Ritonavir - Voriconazole | PK - CYP2C induction by Ritonavir |
|  | Tenofovir disoproxil fumarate - Didanosine | PK - Increase didanosine bioavailability |
